# Post-COVID-19 Necrotizing Pneumonia in Patients on Invasive Mechanical Ventilation

**DOI:** 10.3390/idr13030075

**Published:** 2021-09-08

**Authors:** Alicia Hidron, William Quiceno, John J. Cardeño, Gustavo Roncancio, Cristian García

**Affiliations:** 1 Department of Medicine, Division of Infectious Diseases, Universidad Pontificia Bolivariana, Medellín, Colombia; groncancio@gmail.com (G.R.); vesalio21@yahoo.com (C.G.); 2 Department of Medicine, Division of Infectious Diseases, Hospital Pablo Tobón Uribe, Medellín, Colombia; 3 Department of Radiology, Hospital Pablo Tobón Uribe and Clinica Antioquia, Medellín, Colombia; wquiceno@miune.net; 4 Department of Medicine, Division of Infectious Diseases, IPS Universitaria, Medellín, Colombia; johncardeno@hotmail.com; 5 Department of Medicine, Division of Infectious Diseases, Clinica Cardio VID, Medellín, Colombia; 6 Department of Medicine, Division of Infectious Diseases, Clinica Universitaria Bolivariana, Medellín, Colombia

**Keywords:** necrotizing pneumonia, critically ill, COVID-19, mechanical ventilation, pulmonary embolism

## Abstract

(1) Background: Few reports of necrotizing pneumonia in patients with COVID-19 have been published. We have observed an elevated incidence at two hospitals in our city, suggesting this complication is not uncommon, and may have been overlooked. (2) Methods: This article presents a retrospective, descriptive cohort study that was undertaken from 22 March 2020 to 15 June 2021 in two tertiary care hospitals in Medellín, Colombia. All adult patients admitted to the intensive care unit (ICU) for respiratory failure related to confirmed COVID-19, on invasive mechanical ventilation (IMV), with imaging or surgical findings documenting necrotizing pneumonia (NP) were included. (3) Results: Of 936 patients with COVID-19 that required IMV, 42 (4.5%) developed NP. Overall mortality was 57% and in-hospital mortality was 71%, occurring 15–79 days after COVID-19 diagnosis. NP was diagnosed at a median of 27 days after COVID-19 symptom onset and 15.5 days after initiation of IMV. Infections were polymicrobial in 52.4% of patients. *Klebsiella pneumoniae* (57%) and *Pseudomonas aeruginosa* (33%) were the most common etiologic agents. Pulmonary embolism (PE) was documented in 13 patients overall (31%), and in 50% of patients who underwent an angioCT. Drainage and/or surgical procedures were performed on 19 patients (45.2%) with a 75% mortality rate. (4) Conclusions: In our experience, NP is a relatively common, albeit neglected, complication in mechanically ventilated COVID-19 patients, possibly originating in poorly vascularized areas of lung parenchyma. Associated mortality is high. Although drainage procedures did not seem to favorably impact patient outcomes, diagnosis and treatment were late events in the overall disease course, suggesting that early recognition and timely treatment could have a positive impact on prognosis.

## 1. Introduction

Necrotizing pneumonia (NP) has usually been described in the setting of community-acquired pneumonia (CAP) as a rare complication. Precise data on the frequency of this complication is lacking, but it has been reported in less than one percent of cases of pneumonia, although CT evidence of NP may be retrospectively reported in up to twelve percent of cases of CAP [1]. Disease spectrum includes necrosis of lung parenchyma with abscess formation, cavitation, and gangrene. Pulmonary embolism (PE) has been associated with the development of NP, and may be the common preceding event [2], with infection of infarcted areas of the lung. Although treatment consists of prolonged courses of antibiotics, antibiotic penetration into necrotic areas of the lung is suboptimal, leading to progressive destruction of lung parenchyma and perpetuating the infectious process; thus, surgical treatment may be necessary for cases failing medical management [3]. Although the mortality of necrotizing pneumonia has not been clearly reported in the literature, the survival rate of lung resection for necrotizing pneumonia refractory to medical therapy is extremely elevated (ranges between 80% and 95%), and sequelae may be frequent for those who survive [1,4]. Scant data are available on NP in patients with COVID-19. We report the first series of 42 cases of necrotizing pneumonia in mechanically ventilated patients with COVID-19, with cases arising from two referral hospitals in Medellín, Colombia.

## 2. Materials and Methods

We conducted a retrospective study from 22 March 2020 to 15 June 2021 in two hospitals in Medellín, Colombia: Hospital Pablo Tobón Uribe (HPTU) and Clinica Cardio Vid (CCV). Both hospitals are private, tertiary care, referral hospitals (HPTU is 494 beds, with 102 critical care beds during the pandemic; CCV has 130 beds, with 36 intensive care unit (ICU) beds during the pandemic). All adult patients admitted to the ICU for respiratory failure related to confirmed COVID-19 (with a positive RT-PCR for SARS CoV-2), on invasive mechanical ventilation (IMV), with imaging or surgical findings documenting NP, were included. Diagnosis of NP was performed either by chest computed tomography (CT) documenting pneumonic consolidation with areas of low attenuation (necrosis), with or without pneumatocele(s), abscess(es), or cavitation(s) in the areas of consolidation [4], or by documentation of pulmonary necrosis, gangrene, or pneumatocele(s), with or without pneumatocele(s), abscess(es), or cavitation(s) in the areas of consolidation at the time of surgery. Data were collected on patient demographics, laboratory and radiologic studies, treatment, and outcomes. Categorical variables are presented as absolute frequencies and percentages. Continuous variables are presented as means and standard deviations or medians and interquartile ranges according to normal distribution. Analyses were performed using Stata/IC software, version 16 (StataCorp LLC., College Station, TX, USA). The two participating hospitals’ ethics committees approved this study.

## 3. Results

Of 936 patients with COVID-19 admitted to the ICU that required IMV between March 2020 and June 2021 (826 in HPTU, 110 in CCV), 42 (4.5%) developed NP (35 in HPTU, 7 in CCV). Overall mortality for patients on IMV in this cohort was 57% (24 of 42 patients), 21 of 35 patients in HPTU (60%) and 3 of 7 patients in in CCV (43%); however, 3 of the 7 patients (43%) from CCV were still admitted at the time this report was drafted in contrast to only 5 of 35 patients in HPTU (14%). Therefore, in-hospital mortality data was available for 34 of 42 patients (81%); 71% of patients with NP expired within 15–79 days after COVID-19 diagnosis. (Table 1) Overall mortality of COVID-19 patients on IMV at both of the participating institutions was 44% for HPTU and 66.99% for CCV.

In terms of patient characterizations, 83% of patients were male, and patients had a mean age of 61 years. NP was diagnosed 9–65 days (median 27 days) after COVID-19 symptom onset and 1–45 days (median 17.5 days) after initiation of IMV. Of note, 78.6% of patients had risk factors for COVID-19 disease severity, with obesity representing the most common risk factor (40.5%), followed by hypertension (38%) and diabetes (28.6%) (Table 1).

Infections were polymicrobial in 52.4% of patients. *Klebsiella pneumoniae* (57%) and *Pseudomonas aeruginosa* (33%) were the most frequent etiologic agents, followed by *Serratia marcescens* (11.9%), *Proteus mirabilis* (9.5%), *Staphylococcus aureus*, and *Burkholderia cepacia*. Associated bacteremia was present in 30% of patients. Repeat identification of the same bacteria despite adequate-spectrum antibiotic treatment was documented in 66.7% of patients, two to six times, with MIC (minimal inhibitory concentration) creeps in 10 cases (24%). All MIC creeps involved cases of *P. aeruginosa* (7 of 10) and *K. pneumoniae* (4 of 10); no MIC creeps were observed for other bacteria. Of the 10 cases where MIC creeps were documented, 8 involved antibiotics such as piperacillin tazobactam and carbapenems, 7 involved cefepime and aztreonam, and 5 involved ciprofloxacin. Until NP diagnosis, patients had received 1–38 days (median 13 days) of adequate-spectrum antibiotic treatment (Table 1).

**Figure 1:**
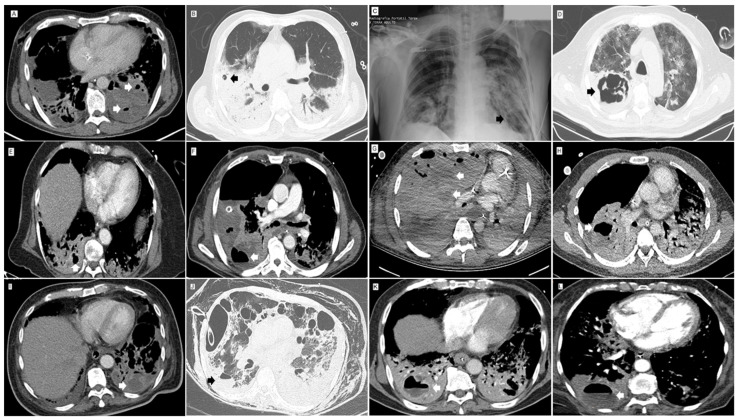
Lung images of post-COVID-19 necrotizing pneumonia. (**A**) Contrast CT. Mediastinal Window. Low density area of the left lower lobe lung parenchyma with loss of parenchymal enhancement, with architectural distortion and small abnormal gas-filled spaces (arrows) due to necrotizing pneumonia. (**B**) Non-contrast CT. Lung Window. Small round 9 mm thin-walled, air-filled lesion (pneumatocele) (arrow) in a right upper lobe area of consolidation. (**C**) Chest X-Ray. Well-defined 20 mm thin-walled area of parenchymal lucency (arrow) within area of consolidation in the left lower lobe. Pneumothorax. (**D**) Non-contrast CT. Lung Window. Thick-walled and irregular cavity (arrow) with septum due to lung abscess within area of necrotizing pneumonia. (**E**) Contrast CT. Mediastinal Window. Heterogeneous attenuation of the right lower lobe lung parenchyma with a consolidation pattern with low-density areas, loss of parenchymal enhancement and small air-filled rounded lesions (arrow) due to necrotizing pneumonia. (**F**) Contrast CT. Mediastinal Window. Large low-density area with loss of parenchymal enhancement of the right upper lobe lung parenchyma associated with thin-walled air-fluid level cavity due to necrotizing pneumonia with lung abscess. Large air-fluid level (arrow) in the pleural space due to empyema. (**G**) Contrast CT. Mediastinal Window. Large area with increase in parenchymal attenuation with a consolidation pattern and low-density areas (arrows), loss of parenchymal enhancement, architectural distortion, and peripheral air-filled rounded lesions due to necrotizing pneumonia. (**H**) Contrast CT. Mediastinal Window. Low-density area with loss of parenchymal enhancement in the right lower lobe, associated with adjacent cavitary lesion (arrow) due to necrotizing pneumonia and lung abscess. (**I**) Contrast CT. Mediastinal Window. Area of consolidation in the left lower lobe with low-density areas (arrow) of liquid content with subtle peripheral enhancement due to necrotizing pneumonia with abscess. (**J**) Non-contrast CT. Lung Window. A 3-cm thin-walled collection with air-fluid level (arrow) due to superinfection of a lung cavity or abscess in the right lower lobe. (**K**) CT pulmonary angiography. Mediastinal window. Bilateral lower lobe consolidation and collection with air-fluid level (arrow) with peripheral enhancement in right lower lobe due to lung abscess. (**L**) CT pulmonary angiography. Mediastinal window. Collection with air-fluid level (arrow) with low peripheral enhancement in right lower lobe consolidation due to lung abscess.

At the time of admission, patients had elevated D dimer (median 1329 ng/mL) and lactate dehydrogenase (LDH) levels (mean 541.8 U/L). At the time of NP diagnosis, patients were hypoxemic (median PaFiO_2_ ratio 138.5), had elevated white blood cell counts (mean 14,161 cells/mm^3^) and C-reactive protein levels (mean 20 mg/dl), and were lymphopenic (median 803.5 cells/mm^3^). For sixteen patients who had follow-up D-dimer measurements, elevations 2–171 times above admission levels were documented. PE was documented in 13 patients overall (31%), and in 13 of 26 (50%) patients who underwent an angioCT. Pulmonary infarctions were documented in eight patients overall, four of which did not have a documented PE. Aside from consolidations with or without evidence of necrosis, the most frequent tomographic and/or surgical findings included the following: pulmonary abscess(es) (50%), mediastinal and/or hilar adenopathies (47.6%), cavities (40.5%), pneumatoceles (38%) and pneumothorax (35.7%) (Table and Figure 1).

Drainage and/or surgical procedures were performed on 19 patients (45.2%). Excluding the patients that were still admitted (*n* = 8), 12 of 16 of patients who underwent surgical procedures died (75%), versus 12 of 18 patients who did not (66.7%) (Table 1). Of the 19 drainage procedures, 9 involved lobectomy and/or decortication, 6 involved percutaneous drainage, and the remaining 4 patients underwent both surgical and percutaneous drainage procedures. For the 16 patients with available mortality data (patients who were not still admitted), 5 of 8 patients (62.5%) who underwent a lobectomy and/or decortication, 4 of 5 (80%) patients who underwent percutaneous drainage, and 3 of 3 (100%) patients who underwent both types of drainage procedures, expired.

## 4. Discussion

Necrotizing pneumonia (NP) has been primarily described in the setting of community-acquired pneumonia (CAP), as a rare and severe complication [1,4]. Data on its overall prevalence is lacking; it has been reported as a complication seen in less than one percent of adult patients with CAP, although it may be a more frequent tomographic finding [1]. A recent review reported only five cases during a six-year period [3]. It is also an infrequent complication in COVID-19 patients, with only a few reports to date [5,6,7]. Except for one case of NP reported in a cohort of 298 patients with COVID-19 pneumonia (less than 0.3%), the incidence of this complication in COVID-19 patients is unknown [8]. Here, we report 42 NP cases over a 14-month period in 913 COVID-19 patients on IMV, for an incidence of 4.5%. This is, to our knowledge, the highest incidence of NP reported to date and the first cohort of patients with NP in COVID-19.

The spectrum of necrotizing lung infections ranges from lung abscess formation to necrotizing pneumonia and finally, pulmonary gangrene [3,9]. Chest X-rays have a low sensitivity for detecting necrosis; thus, a chest CT scan is usually required for diagnosis, which may show patchy or diffuse consolidation in multiple lobes of the lung, destruction of lung parenchyma, loss of parenchymal enhancement, cavities, or lung abscesses [1,4]. Initially, multilobar involvement and ground-glass opacities (GGO) with a rounded morphology, and peripheral distribution, were described as typical CT findings in COVID-19 patients; less commonly, a consolidation or a crazy-paving pattern were described. However, lung cavitation was described to be notably absent [10]. More recently, pulmonary abscess [2,8,11,12,13], infected pneumatoceles [14], and lung cavitation [11,15,16,17,18,19,20] have been described in COVID-19 patients. Aside from areas of consolidation with necrosis, characteristic of NP, radiologic and/or operative findings in our cohort included predominantly pulmonary abscess(es), mediastinal and/or hilar adenopathies, cavities, pneumatoceles, and pneumothorax.

As the disease progresses within the NP spectrum, the bronchial and pulmonary vascular supply is compromised, leading to progressive devitalization of lung parenchyma, impeding adequate delivery of antibiotics to areas of under-perfused tissue, fostering an adequate environment for uncontrolled infection, and causing further tissue destruction [1,3]. Preceding PE may lead to lung infarction, cavitation, abscess formation, and bronchopleural fistula formation [2]. There is a widely reported association between COVID-19 and high thrombotic risk [21], and lung cavitation following PE has been reported in at least one COVID-19 patient [20]. In fact, thrombi in small arterial vessels of the pulmonary vasculature have been documented as a frequent post-mortem finding in autopsy studies [22]. In our cohort, PE was documented in only 31% of patients overall; however, it was documented in 50% patients who underwent an angioCT. Furthermore, pulmonary infarctions were documented in 50% of patients who did not have a documented PE, and D-dimer elevations were documented in 85% of patients who had this test performed, with elevations that were 2 to 171 times above those at admission, suggesting that some thrombotic events may go unrecognized. Thus, pulmonary vasculature thrombosis, associated lung infarction, bacterial colonization of poorly vascularized parenchyma, and subsequent infection, appear to be the common underlying events [2].

*Streptococcus pneumoniae* and *Staphylococcus aureus* (*S. aureus*) have been described as the most common etiologic agents in NP [3,4]; however, *K. pneumoniae* and *P. aeruginosa* are more frequently associated when pulmonary gangrene is present [3]. In this cohort, over 50% of infections were due to *K. pneumoniae,* over one-third were explained by *P. aeruginosa,* close to 20% were due to *Serratia marcescens* and *Proteus mirabilis*, and the remaining infections were due to *S. aureus*, *B. cepacia*, and *E. cloacae*. The majority of infections were polymicrobial (52.4%), and of these, eight (36.4%) included *Candida*, *Aspergillus* spp., or other mold infections. The polymicrobial nature of these infections may be related to NP presenting as a late phenomenon (median of 27 days after COVID-19 symptom onset), perhaps allowing for ongoing colonization in areas of devitalized lung tissue where adequate delivery of antibiotics is limited, fostering an adequate environment for uncontrolled polymicrobial infections. The clinical presentation involved a persistent inflammatory response syndrome, in some cases with repeated isolation of the etiologic agent, that failed to respond to treatment despite a median of 13 days of appropriate-spectrum antibiotics for the isolated bacteria, in some cases with recurrent isolation of the etiologic agent.

There is no consensus on whether a surgical intervention is required nor has there been a proposal regarding the optimal timing for an intervention. Percutaneous drainage is a treatment alternative for a solitary lung abscess, but it may have deleterious effects in necrotizing pneumonia [9,23]. In cases failing medical management, open surgical debridement of devitalized tissue may be necessary [9,23]. In our cohort, 45.2% of patients underwent drainage and/or surgical procedures, with a 75% mortality rate observed, which was similar to a 66.7% mortality rate for patients who did not have a drainage procedure performed. Thus, drainage procedures did not seem to impact mortality in this cohort of patients. 

Mortality in this cohort was 71%, higher than that reported for cohorts of non-COVID-19 NP (40%) [3], and higher than the overall mortality of COVID-19 patients on IMV at both of the participating institutions (44% for HPTU and 67% for CCV). Although mortality for patients who underwent surgical procedures was elevated (75%), it seems to have been lower than that reported for a similar group of patients in the literature (80–95%). Whether this represents the timing of the surgery, patient selection, surgical expertise, or a combination of these factors remains to be determined. Reasons for the overall elevated mortality in NP patients are still elusive but may be related to a delayed diagnosis and elevated underlying disease severity, given that clinical presentation of NP is a late phenomenon in the overall disease spectrum.

In conclusion, NP is a common, probably overlooked, complication in mechanically ventilated COVID-19 patients, which may be directly related to mortality in the late phases of disease. NP should be suspected in patients with a persistent systemic inflammatory response syndrome that fails to respond to pathogen-guided antibiotic therapy, especially if presenting after 15 or more days of IMV, in patients with a preceding PE or increasing levels of D-dimer. Although there is not enough data to assess whether medical therapy alone versus medical therapy plus drainage procedures impacts mortality, what is clear is that associated mortality is high regardless of the treatment strategy. However, compared to historical mortality data in patients who underwent lung resection for necrotizing pneumonia refractory to medical therapy, a lower mortality was observed, suggesting that early recognition and treatment might impact mortality. 

## Figures and Tables

**Table 1:** Demographics, clinical characteristics, and outcomes of patients with COVID-19 on invasive mechanical ventilation with necrotizing pneumonia.

Hospital *n* (%)	
Hospital Pablo Tobón Uribe (HPTU)	35 (83.3)
Clinica Cardiovid (CCV)	7 (16.7)
Mean age—years (SD)	61 ± 13
Male sex *n* (%)	35 (83.3)
Year of admission *n* (%)	
2020	20 (47.6)
2021	22 (52.4)
Median days from first symptom to dx (IQR)	27 (22–32)
Median days of IMV to dx (IQR)	15.5 (11–23)
Comorbidities *n* (%) *	
Obesity	17 (40.5)
Hypertension	16 (38.1)
Diabetes	12 (28.6)
Current/former smoker	8 (19.0)
Immunosuppression	4 (9.52)
CKD	2 (4.76)
COPD	1 (2.38)
CAD	1 (2.38)
No comorbidities	9 (21.43)
Etiology *n* (%)	
*K. pneumoniae*	24 (57.14)
*P. aeruginosa*	14 (33.33)
*S. marcescens*	5 (11.9)
*P. mirabilis*	4 (9.52)
MSSA	3 (7.14)
*B. cepacia*	2 (4.76)
MRSA	1 (2.38)
*E. cloacae*	1 (2.38)
*Candida, Aspergillus* spp., or other molds (%)	8 (19.0)
Bacteria isolated ≥ 2 times *n* (%)	28 (66.67)
MIC creep *n* (%)	11 (26.19)
Associated bacteremia *n* (%)	12 (30)
Polymicrobial etiology *n* (%)	22 (52.38)
Median antibiotic days prior to dx (IQR)	13 (7–17)
Clinical findings	
Median D Dimer at admission (IQR) ng/mL	1329 (713–3621)
Mean LDH at admission U/L	541.81 ± 197.57
Median PaFiO_2_ ratio at dx (IQR)	138.5 (118–172)
Mean WBC count at dx cells/mm^3^ (SD)	14161 ± 4845
Median lymphocyte count at dx cells/mm^3^ (IQR)	803.5 (500–1100)
Mean CRP at dx mg/dl (SD)	20.06 ± 9.43
Imaging and/or surgical findings *n* (%) *	
Abscess	21 (50.0)
Mediastinal/hilar adenopathies	20 (47.62)
Cavity	17 (40.48)
Pneumatocele	16 (38.1)
Pneumothorax	15 (35.71)
Pulmonary embolism	13 (30.95)
Bronchopleural fistula	11 (26.19)
Empyema	9 (21.43)
Pulmonary infarction	8 (19.05)
Surgical/drainage procedure *n* (%) *	19 (45.24)
Percutaneous drainage *n* (%)	10 (23.81)
Decortication *n* (%)	10 (23.81)
Lobectomy *n* (%)	9 (21.43)
Median number of procedures per patient (IQR)	1 (1–2)
Outcome *n* (%)	
Death (overall)	24 (57.14)
Death (excluding patients still admitted)	24 (70.58)
Median days from dx to death (IQR)	37 (23.5–43)
Hospital discharge	10 (23.81)

Table 1 Legend. SD: standard deviation; *n*: number; dx: diagnosis; IQR: interquartile range; IMV: invasive mechanical ventilation; COPD: chronic obstructive pulmonary disease; CKD: chronic kidney disease; CAD: coronary artery disease; *K. pneumoniae*: *Klebsiella pneumoniae*; *P. aeruginosa*: *Pseudomonas aeruginosa*; *S. marcescens*: *Serratia marcescens*; *P. mirabilis*: *Proteus mirabilis*; MSSA: methicillin-sensitive *Staphylococcus aureus*; MRSA: methicillin-resistant *Staphylococcus aureus*; *B. cepacia*: *Burkholderia cepacia*; *E. cloacae*: *Enterobacter cloacae*; MIC: minimum inhibitory concentration; LDH: lactate dehydrogenase; PaFiO_2_ ratio of arterial oxygen partial pressure (PaO2 in mmHg) to fractional inspired oxygen (FiO2); WBC: white blood cell; CRP: C-reactive protein. * The sum may exceed 42 because each case may have had one or more of the characteristics in the list.

## Data Availability

Data can be found in an Excel database at the main researcher’s institution.
